# Impact of Sex Differences and Diabetes on Coronary Atherosclerosis and Ischemic Heart Disease

**DOI:** 10.3390/jcm8010098

**Published:** 2019-01-16

**Authors:** Rosalinda Madonna, Carmela Rita Balistreri, Salvatore De Rosa, Saverio Muscoli, Stefano Selvaggio, Giancarlo Selvaggio, Péter Ferdinandy, Raffaele De Caterina

**Affiliations:** 1 Center of Aging Sciences and Translational Medicine—CESI-MeT, Institute of Cardiology, “G. d’Annunzio” University, Via dei Vestini 31, 66100 Chieti, Italy; 2 Department of Internal Medicine, University of Texas Medical School in Houston, Houston, 77065 TX, USA; 3 Department of Biomedicine, Neuroscience and Advanced Diagnostics (Bi.N.D.), University of Palermo, 90134 Palermo, Italy; carmelarita.balistreri@unipa.it; 4 Division of Cardiology, Department of Medical and Surgical Sciences, University “Magna Græcia’’ of Catanzaro, Viale Europa, 88100 Catanzaro, Italy; saderosa@unicz.it; 5 Department of Cardiovascular Disease, Tor Vergata University of Rome, 00133 Rome, Italy; saveriomuscoli@gmail.com; 6 Geriatric Division, A.R.N.A.S. Ospedale “Garibaldi” Nesima, 95122 Catania, Italy; stefanosel@yahoo.it; 7 Studio Medico Specialistico “VITTORIA”, 97019 Vittoria, RG, Italy; gialose@hotmail.it; 8 Department of Pharmacology and Pharmacotherapy, Semmelweis University, 1085 Budapest, Hungary; peter.ferdinandy@pharmahungary.com; 9 Pharmahungary Group, 6722 Szeged, Hungary; 10 Institute of Cardiology, University of Pisa, C/o Ospedale di Cisanello, Via Paradisa 2, 56124 Pisa, Italy

**Keywords:** coronary artery disease, gender, sex, women, diabetes, cell signaling, biomarkers

## Abstract

Cardiovascular diseases (CVD) including coronary artery disease (CAD) and ischemic heart disease (IHD) are the main cause of mortality in industrialized countries. Although it is well known that there is a difference in the risk of these diseases in women and men, current therapy does not consider the sexual dimorphism; i.e., differences in anatomical structures and metabolism of tissues. Here, we discuss how genetic, epigenetic, hormonal, cellular or molecular factors may explain the different CVD risk, especially in high-risk groups such as women with diabetes. We analyze whether sex may modify the effects of diabetes at risk of CAD. Finally, we discuss current diagnostic techniques in the evaluation of CAD and IHD in diabetic women.

## 1. Introduction

Cardiovascular diseases (CVD), such as coronary artery disease (CAD), coronary heart disease (CHD, including myocardial infarction) and ischemic heart disease (IHD), are the main cause of mortality in the European Union, accounting for nearly 45% of all deaths in Europe and 37% of all deaths in the European Union in 2017 [1]. Cardiovascular risk in people with diabetes mellitus (DM) is significantly higher than those without the disease. However, increasing evidence indicates that risk of CAD and IHD is different in women and men. Indeed, the relative risk of fatal IHD associated with diabetes is 50% higher in women than men [2]. Furthermore, the diagnosis of CAD is more challenging in women, as traditional diagnostic tests are less sensitive and specific in female patients, showing a lower prevalence of obstructive CAD, a greater burden of symptoms, and a high atherosclerotic burden [3]. Current treatments are limited, as the best available therapy reduces the cardiovascular risk by only 25–30%, and remains virtually ineffective in reducing the excess risk associated with diabetes. In addition, the propensity to treat is significantly lower for women than for men, especially at a younger age. In fact, most cardiovascular treatments, including antiplatelet agents, beta-blockers, lipid-lowering agents or coronary angioplasty were significantly less frequent in women in a recent study involving patients aged 35 to 54 years [4]. Furthermore, current therapeutic approaches do not consider sexual dimorphism between women and men, including differences in anatomical structures (i.e., size and body composition, as well as the different nature of adipose tissue [5]). In line with this assumption, it has recently been shown that sexual dimorphism influences the expression of genes related to the mitochondria of human cells in the body, including cardiovascular cells [6]. Therefore, sexual dimorphism may result in a different pharmacokinetics of the currently used drugs for the treatment of CVD, as well as a different response to them in terms of gene expression and epigenetic differences.

Here, we discuss how genetic, epigenetic, hormonal, cellular or molecular factors may explain the higher risk for CAD in women, especially in high-risk groups such as women with diabetes. We will analyze how sex modulates the effects of diabetes on CAD and IHD. Finally, we discuss the available evidence on the role of current diagnostic techniques in the evaluation of CAD and IHD in diabetic women.

## 2. Coronary Artery Disease and Ischemic Heart Disease in Diabetic Women: Defining the Problem

The cardiovascular risk in people with diabetes is two to three times higher than those without the disease [7]. However, for people without diabetes these estimates assume that diabetes confers the same degree of risk in women as in men, which is unlikely because while non-diabetic women are relatively protected from CVD, this advantage is lost in diabetes [8]. In the Framingham study, the incidence of symptomatic heart failure was 2.4 times higher in men and 5.0 times higher in women with diabetes as compared to non-diabetics [9]. This observation has been confirmed by other studies [10]. In the HANAMI study, which included 764 subjects within the Mediterranean area, the rs146052672 variant of High Mobility Group A 1 (HMGA 1) protein, associated with insulin resistance, type 2 diabetes and the development of CVD was present in 50% female patients with diabetes who had an acute myocardial infarction (AMI) compared to 24% of their male peers [11]. Furthermore, diabetic women have a significantly higher mortality after myocardial infarction than diabetic men [12,13]. There is a three times higher risk for fatal CAD in women with type 2 diabetes as compared to nondiabetic women (95% confidence interval (CI), 1.9–4.8) [14]. Women with type 2 diabetes have a higher adjusted hazard ratio (HR) of fatal CAD (HR = 14.74; 95% CI, 6.16–35.27) compared to type 2 diabetes men (HR = 3.77; 95% CI, 2.52–5.65) [15]. In a meta-analysis of over 850,000 individuals the relative risk of CVD was 44% greater in women with diabetes than in similarly affected men [16].

A gender difference was also observed in pre-clinical diabetic cardiomyopathy. In a series of 100 adults (44% females) with no previous evidence of heart disease, echocardiography showed the presence of diabetic cardiomyopathy in 48% of patients, with the female gender among the strongest predictors of cardiac remodeling [17]. In a series of 80 children and adolescents with well-controlled type 1 diabetes, abnormalities in left ventricular dimensions and myocardial relaxation were reported, with girls clearly more affected than boys [18]. Similarly, the relative risk of diabetes-related CAD is substantially higher in women than in men, even after differences in other major cardiovascular risk factors have been considered [16,19,20]. A recent systematic review and meta-analysis of all prospective data available also estimate the relative effect of diabetes on the risk of stroke significantly higher in women than in men, even after adjustment for differences in other major cardiovascular risk factors [16,21,22]. These data add to existing evidence that men and women experience diabetes-related diseases differently and suggest the need for further work to clarify the biological, behavioral, or social mechanisms involved. In fact, the reason for this global “female disadvantage” in diabetes remains largely unknown. Sexual disparity in the management and treatment of cardiovascular risk factors in individuals with diabetes, is possibly involved [23]. Data of nearly two million individuals with diabetes in the United Kingdom have suggested that women with diabetes were less likely than men with diabetes to receive treatment recommended by national guidelines and to meet treatment targets [24]. These sex disparities in the treatment and management of individuals with diabetes alone, however, may be too small to explain all the relative excess risk for CVD in women with diabetes. Alternatively, as the detrimental effects of glucose have already occurred at glycemic levels below the threshold for diagnosing diabetes, it could be that the transition from normoglycemia, to impaired glucose tolerance and overt diabetes is more detrimental in women than in men [23]. As diabetes can remain undetected for many years (about 183 million people with diabetes, 50% of all people with diabetes, undiagnosed) [7], diagnosis is often made at an advanced stage, especially in women due to disparities in diagnosis and treatment mentioned above, when major metabolic alterations and vascular complications have already occurred in most patients [25]. Accumulating evidence suggests that these adverse changes in metabolic and vascular risk factor profile in pre-diabetic individuals are greater in women than in men [26,27,28,29,30,31,32]. These data may suggest that the diabetes-related increased risk of cardiovascular disease in women may also be due to the combination of both a greater deterioration in cardiovascular risk factor levels and a chronically elevated, but undiagnosed and untreated, cardiovascular risk profile in the pre-diabetic state, beside any significant sex difference in the effects and complications of diabetes itself [33,34].

## 3. Genetic, Epigenetic, and Hormonal Factors Involved in Sex-Specific Effects of Diabetes in CVD

Studies on animal models have helped to elucidate the potential pathogenic mechanisms of CVD, but not to explain the interaction between sex/gender and diabetes, since most of the information available derives from investigations performed on male rodents [35]. Thus, there is not yet sufficient evidence on how gender differences modify the prevalence and incidence of CVD in the context of type 1 and or type diabetes. The mechanisms highlighted so far are essentially linked to the sex hormones like estrogens, with clear influences on the differential regulation of the immune system in both sexes (Table 1).

### 3.1. The Immune System Profile in Women

Women show a different profile of the immune system than men (the so-called immune dimorphism) [36]. Specifically, women have lower proportions and numbers of CD8+ T cells, higher proportions and numbers of CD4+ T cells, and higher CD4/CD8 ratios than men [36]. These sex differences appear to be hormonally mediated since estrogen deficiency increases the proportion and number of CD8+ T cells and decreases the CD4/CD8 ratio [36]. Thus, a lower number of CD8+ T cells could contribute to the high incidence of chronic diseases in women, including metabolic disorders and autoimmune diseases, as well as increased susceptibility to infections, particularly virus infections.

### 3.2. Genetics and Epigenetics

Genetic and epigenetic factors seem to be behind the aforementioned sexual dimorphism. Women have higher expression of HLA-DR3 and DR4 alleles than men, decreased DNA methylation of X-chromosome-related genes, sex chromosome instability, and escape from X-chromosome inactivation [37].

### 3.3. Environmental Factors and Gut Microbiome

Environmental factors, such as pathogens, xenobiotics and smoking, also seem to favor the onset and progression of metabolic and CVD diseases differently in both sexes, likely inducing a sustained activation of immune/pro-inflammatory innate pathways than anti-inflammatory pathways, which is facilitated by lower numbers of CD8+ T cells in women [37]. To this, we must also add the crucial contribution of the gut microbiome, which differs between women and men, and seems to contribute to the sex differences in diabetes and susceptibility to CVD [46,47]. This is consistent with the results of studies reported in the literature in recent years, which emphasize the close relationship between the gut microbiome and human health. Age and sex related perturbations in the gut microbiome are linked to a wide range of conditions, including bowel disorders, autoimmune diseases, obesity, and metabolic disorders [37]. The direct effect of the gut microbiota in obesity has been elegantly highlighted in studies on mice, in which the gut microbiota from lean or obese mice were transplanted into mice without germs. Mice with microbiota from diet-induced or genetically obese mice showed a high weight compared to mice with microbiota from lean donors [44]. These results demonstrate that the gut microbiome dramatically influences the host metabolism, including effects on the acquisition and storage of energy, but also on the activity and phenotype profile of the immune system [37]. Thus, alterations in the gut microbiome can impact the onset of diabetes and CVD in the two sexes differently (Table 1).

### 3.4. Different Metabolism in Women: Correlation with the Different Asset of Sex Chromosome Genes

Dehghan and co-workers demonstrated the effect of inulin ingestion in controlling inflammation and metabolic endotoxemia in women with diabetes [45] (Table 1). The effect of inulin underlines the different metabolism of women, whose understanding is fundamental for the development of appropriate measures for the prevention, diagnosis, and treatment of diseases. Specifically, women have mechanisms that have evolved to favor the storage of adipose tissue. Thus, they tend to accumulate fat mass and body weight and have an increased subcutaneous adipose tissue. In contrast, fat mobilization is more efficient in males. In addition, women tend to have higher insulin sensitivity, and the sexes differ in lipoprotein profiles. These differences are related to the levels of sex hormones, which are reduced after menopause. Accordingly, investigations have demonstrated that post-menopausal women have altered body fat distribution and increased incidence of CVD, hypertension, diabetes, and other disorders [2,39,40]. These differences are not only related to sex hormones, but also to sex chromosome complement. Thus, sex chromosome asset (or better sex chromosome genes asset) can contribute to sex differences in metabolic traits [41,42,43] (Table 1).

### 3.5. Estrogenes and Oxidative Stress

Estrogens also appear to affect the oxidative stress, which is considered a key mechanism in the onset of CVD and diabetes (see Section 4.3 paragraph). Women before the menopause show lower levels of oxidative stress than men, due to the antioxidant properties of estrogens, which modulate the expression and levels of anti-oxidant enzymes, such as nicotinamide adenine dinucleotide phosphate (NADPH)-oxidase (especially p47, Nox levels) and angiotensin II [38].

## 4. Cellular and Molecular Signaling Pathways Involved in Sex-Specific Effects of Diabetes in CAD

**Figure 1:**
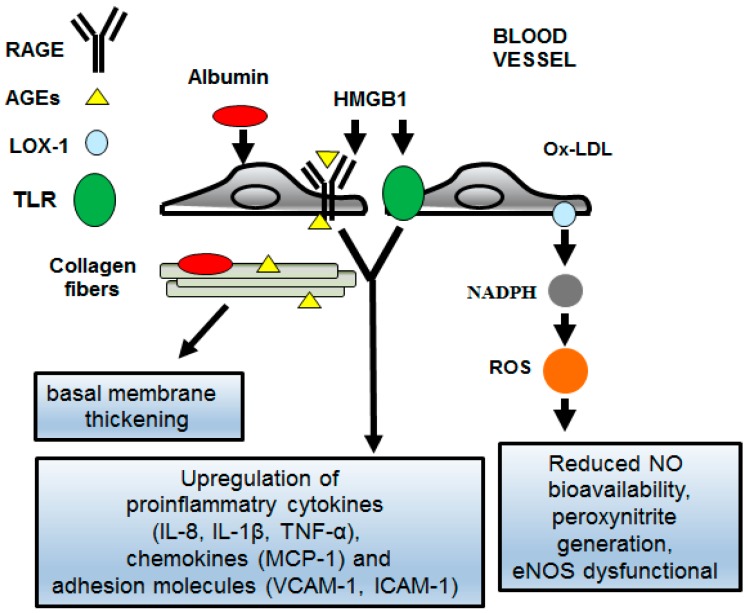
Molecular pathways associated with diabetic macroangiopathy. The figure depicts some of the key points discussed in the text on the role of advanced end products (AGE) products, innate immunity, and reactive oxygen species generation in diabetic macroangiopathy. Abbreviations: RAGE, receptor for advanced glycation end products; AGEs, advanced glycation endproducts; TLR, Toll-Like Receptor; HMGB1, high mobility group box 1; IL, interleukin; TNF-α, tumor necrosis factor alpha; MCP-1, Monocyte chemoattractant protein-1; VCAM-1 vascular cell adhesion molecule 1; ICAM-1, intercellular adhesion molecule 1; ROS, reactive oxygen species; ox-LDL, oxidized low-density lipoprotein; NADPH, nicotinamide adenine dinucleotide phosphate; NO, nitric oxyde; eNOS, endothelial nitric oxide synthase.

A wide range of molecular mechanisms and inflammatory signaling pathways are associated with the increased susceptibility of women to diabetes and CVD, such as CAD, as suggested by the meta-inflammation hypothesis and widely summarized in the recent review of Aravindhan and Madhumitha [48] (Figure 1). Specifically, women are characterized by a unique immune system and metabolic profile, which is responsible for sustained activation of immune/pro-inflammatory innate pathways and for the release of age-specific arterial secretory phenotype (AAASP) [49]. In turn, AAASP further exacerbates the systemic chronic low-grade of inflammation associated with both onset of diabetes and related complications, such as CAD according to the concepts of meta-inflammation [50,51]. Among the AAASP molecules, proinflammatory cytokines and chemokines, and the monocyte chemoattractant protein (MCP-1), contribute to evocate a strong local inflammatory state, and increase the low-grade systemic inflammation [50,51]. In addition, AAASP generate dyslipidemia by increasing the accumulation of triglycerides-enriched lipoproteins directly (especially VLDL) [52].

The latter is also increased by both the excess of insulin and the insulin resistance, which also contribute to the systemic accumulation of free fatty acids (FAs). Moreover, these mechanisms associated with hyperglycemia determine the accumulation of toxic intermediates of lipid metabolism, such as ceramide and diacylglycerol, and the accumulation of advanced end products (AGE). Toll-like receptor (TLR)-2 and -4 [48], and receptors for advanced glycation end products (RAGEs), mediate the biological effects described above by inducing independent responses or cross-mediated actions via an active cross-talk between the two types of receptors. AGEs recruit inflammatory molecules by interacting with RAGE, and elicit oxidative stress by increasing the production of reactive oxygen species (ROS) and subsequently evoke the proliferation and fibrotic reaction. Thus, TLR-4 plays a key role in the onset and progression of atherosclerosis, as well, as in CAD and other inflammatory diseases, such as diabetes [53]. Moreover, it has recently been suggested that the crosstalk between TLR-4 and dipeptidyl peptidase-4 (DPP-4)-incretin system is significantly associated with diabetes and CAD. The whole action of all these signaling pathways, which is depicted in Figure 1, causes changes in the coronary artery wall, including endothelial disruption, intima-media thickening, arterial amyloidosis, fibrosis, elastin fracture, glycoxidative changes of the matrix, and the calcification [53,54,55].

### 4.1. The AGEs/RAGEs System in Diabetic Women

AGEs are formed during the Maillard process, a non enzymatic reaction between reducing sugars and amino groups of proteins, lipids and nucleic acids [56]. The formation and accumulation of AGEs occur at sustained rates in diabetic patients, and represent one of the most important mechanisms of the pathophysiology of diabetic CAD. AGEs mediate their effects through the RAGE receptor, a multi-ligand receptor and member of the super-family of immunoglobulin cell surface molecules, expressed in vascular cells. They include NF-κB activation, the increase in the expression of cytokines and adhesion molecules, the induction of oxidative stress, and the increase in the formation of cytosolic reactive oxygen species (ROS) [56]. Furthermore, AGEs cause chemical and biophysical changes in the collagen structure of the extracellular matrix, leading to functional alterations; i.e., thickening of the basal membrane and increased resistance to proteolytic digestion [56]. In diabetic women, RAGE is also induced by other ligands, such as high mobility group box 1 (HMGB1) that can interact with both RAGE and TLR4 (and TLR2). This increases the activation of NF-κB and the expressions of proinflammatory cytokines and pro-angiogenic molecules. AGEs have different intra- and extracellular targets, so they can be considered as a “bridge” between intracellular and extracellular damage. Moreover, whatever the level of hyperglycemia, AGE-related intracellular glycation of mitochondrial respiratory chain proteins has been found to produce more abundant ROS, which further promotes the formation of AGEs. The excessive formation of AGE leads to the thickening of macrovessel, hypertension, endothelial dysfunction, loss of pericytes, decreased platelet survival and increased platelet aggregation. All these abnormalities can promote the procoagulant state, resulting in ischemia and induction of growth factors, with angiogenesis and neovascularization [56] (Figure 1).

### 4.2. The TLR-2 and -4 Signaling Pathways in Diabetic Women

The synergistic interaction between diabetes and CAD in women occurs through sterile inflammation, also known as metabolic inflammation or meta-inflammation [48], by activating TLR-2 and -4 signaling pathways [57]. This is supported by a recent study that showed increased expression of the two TLR pathways in postmenopausal women with metabolic syndrome (MetS) compared to women without MetS, accompanied by an increase in the levels of proinflammatory molecules [58]. On the other hand, the TLR-2 and -4 signaling pathways are expressed in the gut cells, β pancreatic cells and coronary artery cells [59]. In women, changes in the composition and levels of the intestinal microbiota, with advanced age and as an effect of diet, can reduce the integrity of the intestinal barrier and increase the loss of lipopolysaccharides and fatty acids, which can activate TLR-2 and -4 and consequently can induce systemic inflammation. In fact, high levels of several circulating inflammatory molecules are a common feature in the natural course of diabetes [59]. Fatty acids can also trigger endoplasmic reticulum stress, which can be further stimulated by cross talk with active TLR- 4 [57]. Furthermore, recent evidence also suggests that endogenous ligands (i.e., endogenous damage-associated molecular patterns—DAMPs), such as saturated fatty acids and necrotic cellular products, induce the activation of TLR-2 and 4 expressed on the *β*-cells and resident macrophages, leading to insulin resistance, pancreatic *β*-cell dysfunction, and alteration of glucose homeostasis [59]. Furthermore, the cooperation between RAGE and TLR2/and -4 has recently been suggested to coordinate and regulate immune and inflammatory responses in diabetic patients [56]. The synergistic activation of RAGE and TLR2/and-4 leads to the amplification of inflammatory responses. RAGE and TLRs share several common ligands including HMGB [56]. RAGE also appears to interact with TIRAP and MyD88, both of which are intracellular adapter proteins used by TLRs to activate the downstream signaling pathway. The strong interaction between HMGB1-TLR-RAGE triggers the activation of NF-κB and the consequent up-regulation of pro-inflammatory and pro-atherosclerotic genes [56] (Figure 1). Thus, the synergistic action of two pathways, the AGEs/RAGEs axis and the TLR-2 and -4 signaling pathways can play a major role in the atherosclerotic process in diabetic women. The inhibition of these pathways could mitigate both the diabetic and atherosclerotic process and reduce the risk of diabetic CAD. Numerous studies have shown that certain types of cardiovascular drugs, such as statins, angiotensin II receptor antagonists and insulin sensitizers such as rosiglitazone may exert anti-atherosclerosis effects, targeting these signaling pathways [53,54,55].

### 4.3. Oxidative Stress and Ox-LDL in Diabetic Women

Lower concentrations of very low density lipoproteins (VLDL) and low density lipoproteins (LDL) in plasma in women compared to men are associated with accelerated (rather than reduced) VLDL and LDL production [60]. In contrast, the secretion rate of VLDL apolipoprotein B-100 (VLDL particles) is lower in women than in men. As a result, women produce less (on average) triglyceride-richer VLDL than men [61]. There is no information regarding possible sex differences in cholesterol kinetics in the various lipoprotein fractions in human subjects. However, animal studies support the important role of endogenous sex hormones in mediating cholesterol metabolism in a sexually dimorphic manner [62].

In diabetic women, the production of accelerated LDL is accompanied by an increase in lipid peroxidation and by the accumulation of oxidized low-density lipoprotein (ox-LDL) in the intima of the arteries, which are among the main causes of endothelial dysfunction and early atherosclerosis [63,64,65,66]. The accumulation of ox-LDL in the intima and its subsequent uptake by the macrophages, lead to the formation of foam cells through the interaction with lectin-like oxidized low-density lipoprotein receptor-1 (LOX-1) receptor. LOX-1 is the most relevant receptor for ox-LDL in vascular endothelial cells, macrophages and activated smooth muscle cells [65], and in diabetic and obese women its expression is particularly high [67,68]. LOX-1 is a type II membrane protein with a C-type lectin-like structure at the C-terminus, responsible for binding, uptake, and degradation of the ox-LDL. Its expression is highly modulated by AGEs, cytokines, angiotensin (Ang) II, ox-LDL, shear stress and oxidative stress by the activation of NF-κB [69,70,71]. The complete signaling pathway of ox-LDL/LOX-1 in diabetic women is not yet clear. One of the hypothesized mechanisms is that the ox-LDL binding with LOX-1 activates the NADPH oxidase enzyme system, resulting in excessive superoxide generation [72]. The scavenging of nitric oxide (NO) by superoxide can not only reduce the bioavailability of NO, but also generate a more powerful oxidant such as peroxynitrite. Moreover, oxidative stress also downregulates the endothelial nitric oxide synthase (eNOS), which becomes dysfunctional, promoting superoxide formation (Figure 1). Other important mechanisms investigated are the activation of both the polyol and protein kinase C pathways [73]. A recent study evaluated the levels of anti-ox LDL antibodies in 391 women of advanced age [71]. The population was classified as normal or with impaired glucose tolerance, reduced fasting blood glucose or type 2 diabetes (T2DM), according to baseline glycemic levels and after an oral glucose tolerance test. The same patients were studied again six years later. Logistic regression analysis showed that the body mass index (*p* < 0.001) and the levels of anti-ox-LDL antibodies (*p* < 0.04) are among the main independent variables that predict the development of T2DM [71].

## 5. Non-Invasive Assessment and Biomarkers of Coronary Artery Disease in Diabetic Women

**Figure 2:**
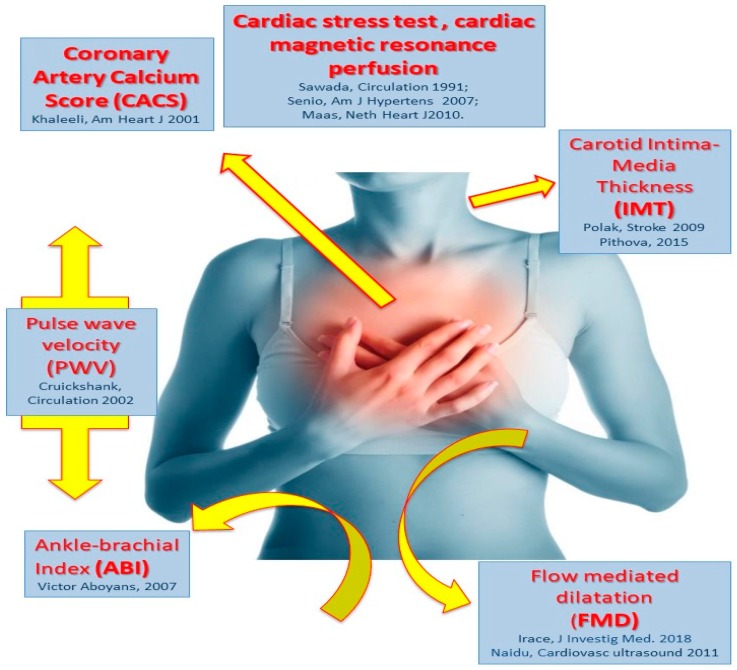
Noninvasive assessment of coronary artery disease in diabetic women. Although the risk of heart disease in diabetic women may emerge before menopause, detection and evaluation of CAD are difficult, due to the lower sensitivity and specificity of biomarker assays in women.

One of the major challenges in the management of women with CAD, especially if diabetic, is that they often present “atypical” symptoms. This is commonly associated with late or missed diagnosis [3]. Still worse, in the acute setting, women usually refer a less defined clinical presentation, including dyspnea, stomach pain, nausea or fatigue, very often without classical chest pain [74]. This may lead to a dangerous delay in the diagnosis [75]. In addition, as females were less represent than men in large biomarker validation studies, standard diagnostic tests do not perform in women as well as they do in men. In fact, according to a recent Scottish study, the usual diagnostic threshold for standard troponin assays was too high for women, and the use of high-sensitivity troponin assays could partially attenuate this shortcoming [76]. In this regard, the most appropriate way to improve diagnostic efficacy in women should be to establish and validate an independent threshold. Women often have a different response to drug treatments than men and this also applies to antiplatelet agents. Low responsiveness in women can be even more dangerous than men, as they usually have a higher residual platelet activity, both at baseline and under antiplatelet treatment [77]. This becomes a key issue in diabetic women with CAD, especially in the context of acute coronary syndromes, also because using aggregometry is less reliable in women than in men. Similar shortcomings are more evident with the use of non-invasive assessment methods. In fact, despite the evidence that more than half of the patients with CAD who die suddenly had no previous clinical evidence of CVD at all, the establishment and robust validation of noninvasive methods to assess subclinical atherosclerosis has always been a challenge [78]. Sudden cardiac death is an eminent exception to other cardiovascular diseases, as it has a higher incidence in women compared to men [79]. As already described above for chest pain, this is partly due to the wrong perception that women are “protected” from CVD and partly to the often atypical clinical presentation of the disease in women. In fact, women with Brugada Syndrome are more frequently asymptomatic and less frequently presenting a spontaneous ECG pattern, compared to men [80]. Unfortunately, despite diabetic women having a high prevalence of preclinical atherosclerosis, the utility of noninvasive screening in women is even more limited, because of a lower sensibility of their men’s counterpart [81]. One common pitfall of current clinical protocols for selection of subjects with suspected stable coronary artery disease is the frequent number of coronary angiographies showing no critical coronary stenoses, thus exposing patients to unnecessary procedural complications, which occurs more often in women than in men [82,83]. Interestingly, using a recently developed novel application of flow-mediated dilation (FMD) that takes into account not only the degree of dilation but the kinetics of its onset showed that the delayed onset of FMD after the induction of ischemia was able to predict the presence of critical coronary stenoses, thus reducing the number of unnecessary negative coronary angiographies in women [84] (Figure 2).

Results of the National Health and Nutrition Examination Survey (NHANES) and Atherosclerosis Risk in Communities (ARIC) showed that the prevalence of an ankle brachial index (ABI) <0.90 in women aged 40 to 59 years was about twice as high as for age-matched men, suggesting different ABI thresholds for the diagnosis of peripheral artery disease (PAD) in men and women. In fact, the mean ABI value in women was about 0.02 lower than in men. Interestingly, despite different thresholds were used in the ARIC study for women (<0.90) and men (<0.08), a higher prevalence of PAD was found in women [85]. Further studies have shown that diabetes is strongly associated with PAD in women. In addition, women with an ABI <0.9 showed a greater extent of inducible myocardial ischemia than those with an ABI ≥0.9, whereas the same was not found in men. This finding is consistent with the more deleterious effect of diabetes on cardiac heart disease development observed in women [15]. Hence, ABI measurement could be useful in risk stratification of diabetic women to be selected for coronary angiography [86]. On the contrary, stress testing studies performed with cardiac imaging demonstrated less extensive ischemia in asymptomatic diabetic women compared with men [87]. However, despite smaller perfusion defects, prognosis of diabetic women is worse compared with men [88]. The absence of coronary artery calcium (CAC) has a very high negative predictive value (99%) for CAD in women above 50 years of age at intermediate cardiovascular risk [89]. Therefore, calcium scoring with multi-slice dual energy computed-dual energy computed tomography (CT) represents a very useful modality to rule out the presence of obstructive CAD, even though the radiation exposure makes this technique less suitable in premenopausal women and for follow-up purposes. Prevalence and severity of CAC in asymptomatic diabetics are similar to that of patients with established CAD but without diabetes. Interestingly, men and women with diabetes have a similar extent of CAC, confirming the clinical evidence that diabetes overrides the usual protection from CVD of women [90]. Positron emission tomography (PET) and cardiovascular magnetic resonance (CMR) are the most sensitive diagnostic methods to detect CAD without obstructive lesions or microvascular disease, both conditions being more prevalent in women than in men [2]. PET might be very helpful to overcome some of the difficulties in the diagnosis of CAD in diabetic patients and especially in women. In fact, it might be very useful in identifying individuals with at increased risk for vascular complications at an early stage, even before they develop symptoms, as functional abnormalities are usually present way earlier than morphological vessel alterations in CAD [91,92,93,94]. The reliable identification of subjects at risk at an early stage would allow the initiation of risk-targeted cardiovascular prevention therapy, with potential impact on the patient’s prognosis [91,95]. Furthermore, PET allows the identification of global or regional circulatory dysfunction, including microvascular disease, through the assessment of myocardial blood flow (MBF). Testing the changes in MBF in response to vasomotor stress (i.e., using the cold pressor test or pharmacological stimuli) might be exploited to identify early vascular dysfunction as well as to monitor the effect of pharmacological treatments [91,96]. Moreover, the development of advanced imaging modalities, that might be capable of identifying sites of vascular inflammation and/or active atherothrombotic processes through noninvasive methods would be of invaluable help to overcome the current problem that noninvasive biomarkers are less effective in women than in men. Among the most promising techniques, the ultrasmall superparamagnetic iron oxide (USPIO) was initially developed for magnetic resonance imaging (MRI), but the advent of the higher resolution dual energy computed tomography (CT) allows to resolve USPIO in atherosclerotic lesions, a reliable marker of activated macrophages [97]. Fusion imaging integrating information from both PET and CT might be very useful in the noninvasive assessment of CAD in women. In fact, multiple diagnostic studies demonstrated that the use of PET/CT scanners is associated with an excellent diagnostic performance in CAD, being able to identify critical coronary stenoses (<70% luminal narrowing) with 92% sensitivity and 90% specificity [98,99]. Combined CT and PET with the radiotracer ^18^F sodium fluoride (^18^F-NaF) injection can identify coronary atherosclerotic plaques that have ruptured or eroded. However, the processes behind ^18^F-NaF uptake in vulnerable plaques remain unclear [100].

Very recently, the use of some nanobody-based radiotracers including vascular cell adhesion molecule (VCAM)-1, macrophage mannose receptor (MMR) and LOX-1 through an integrative multimodality positron emission tomography (PET)/MRI imaging protocol revealed very promising for the noninvasive detection active atherosclerotic processes leading to disease progression or instabilization [101]. This latter approach can be particularly useful for risk stratification in women to overcome the above described limitations of current noninvasive markers, as it has been reported that LOX-1-induced excessive superoxide generation is one factor explaining why high risk female subjects escape from current risk stratification models [72].

Contrast-enhanced MRI might be helpful to identify an otherwise hidden anatomical substrate for SCD risk in women with mitral valve prolapse. In fact, late enhancement was observed in 93% of patients and—most importantly—it was identified within the same region where histopathology substrate for SCD were found [102].

Intima-media thickness (IMT) increases with age, and is usually larger in men than women. Among women with type-1 diabetes mellitus (T1DM), a significant association was also found between common carotid IMT and CAD, measured by intravascular ultrasound [103]. Moreover, IMT was strongly associated with body mass index (BMI), waist circumference, and total body fat in women with T1DM. On the other hand, IMT above 0.9 mm or other signs of preclinical atherosclerosis were associated with dysmetabolic status (fasting glucose, glycated haemoglobin, plasmatic atherogenic index, TG, cholesterol) [104]. To this regard, it should be highlighted that women are exposed to a higher cardiovascular risk after transition to menopause, as a consequence of multiple pro-atherothrombotic changes [105]. Furthermore, women usually gain weight during the menopause, resulting in increased prevalence of the metabolic syndrome [106]. The triglyceride-glucose index (TyG-Index) represents the product of glycaemia and triglyceridaemia and is a simple and useful marker of insulin resistance [107]. A recent study demonstrated that normal-weight post-menopausal women with a TyG-Index crossing the threshold of 8.0 have a significantly higher prevalence of subclinical atherosclerosis as their counterparts with normal TyG index, which might therefore be used as an easy and low-cost screening marker in the lean post-menopausal women [108].

During the last years, initial evidence of specific genetic and epigenetic determinants of cardiovascular risk has emerged. This might be helpful to improve cardiovascular risk stratification in women [109]. For example, Ala16Val (rs4880), the most common gene variant of superoxide dismutase (SOD) 2, a central element of physiological systems that protect cells against free radicals is associated to a higher-than-expected cardiovascular risk in diabetic women, confirming that oxidative stress is pivotal to undercover unapparent cardiovascular risk components [110]. Another example of genetic variants associated with increased cardiovascular risk that is particularly useful in women is the −308 polymorphism of the promoter region of the *TNF-α* gene [111]. A recent genome-wide association study (GWAS) conducted in more than 15,000 women was able to identify eight major pathways that are modulated both by type 2 DM and CVD across different ethnic groups [112].

The clinical evidence and study results reported in this article are shown to highlight the most relevant issues that are currently limiting the management of cardiovascular disease in diabetic women and to raise the awareness on current discrepancies between men and women both on diagnosis and treatment. However, since study designing was largely varying among the studies reported, their results should be interpreted very cautiously. Findings of single studies are not directly comparable to each other and no consequentiality should be assumed between them. In fact, despite adjustment for relevant risk factors is often performed within single studies, it does not imply their generalizability. Moreover, adjustment procedures do not completely exclude concealed influencing factors. Hence all results that are not directing reflecting the main study outcome in randomized studies should be considered hypothesis generating and not conclusive by any means.

## 6. Conclusions

Understanding sex differences and impact of diabetes on epidemiology, clinical presentation, pathogenetic mechanisms and diagnosis of CAD and IHD is important because men and women experience diabetes-related diseases differently, in terms of risk for coronary symptoms prognosis and death. The reason for this global “female disadvantage” in diabetes remains largely unknown. Clearly, the traditional view that the female “advantage” is only due to differences in the sex hormone milieu—in particular, the availability of estrogens is not true, as many other factors can play a relevant role, such as sexual dimorphism, sexual disparity in the management and treatment of cardiovascular risk factors in individuals with diabetes, as well as genetic factors and sex chromosome genes asset. All factors that make the difference in CAD and IHD in diabetic women compared to their male counterparts, in terms of epidemiology, clinical presentation, pathogenesis and diagnosis, are summarized in Table 2. Future studies are needed to determine how sex can modulate the effects of diabetes on cardiovascular risk. Certainly, earlier interventions to better control risk factors for atherosclerotic disease in women with diabetes can be done as it has potential to drastically reduce coronary mortality.

## Figures and Tables

**Table 1:** Mechanisms and Risk Factors for Diabetes in Women.

Mechanisms and Environmental Factors	Risk for Diabetes in Women
A lower proportions and numbers of CD8+ T cells [36]	↑
Viral infections [37]	↑
High expression of HLA-DR3 and DR4 alleles [37]	↑
Decreased DNA methylation of X-chromosome-related genes [37]	↑
Sex chromosome instability [37]	↑
Escape from X-chromosome inactivation [37]	↑
High expression of Receptors for estrogens in the heart and high oxidative stress [38]	↑
Pathogens, xenobiotics and smoking [37]	↑
Different metabolism: High accumulation vs. low mobilization of adipose tissue, and altered distribution of body fat associated with not only sex hormones, but also with sex chromosome complement [39,40,41,42,43]	↑
Microbiome [37,44,45]	↑

Legend: ↑, increased.

**Table 2:** Sex differences and impact of diabetes on coronary atherosclerosis and ischemic heart disease.

	Sex Differences Premenopausal Women vs. Men	Effect of Diabetes Premenopausal Women vs. Men
Cardiovascular risk	↓	↑
Fatal CAD risk	↓	↑
PAD risk	↑	↑
Propensity to treat	↓	↓
Delayed diagnosis	↑	↑
CD4+/CD8+ T cells ratio	↑	~
HLA-DR3 and DR4 alleles expression	↑	~
DNA methylation of X-chromosome-related genes	↓	~
Sex chromosome instability	↓	~
Escape from X-chromosome inactivation	↓	~
Activation of immune/pro-inflammatory innate pathways	↑	↑
Different metabolism: High accumulation vs. low mobilization of adipose tissue	↑	~
Oxidative stress	↓	↑
Intestinal microbiome	↑	↑
AGEs/RAGE activation	~	↑
TLR-2 and -4 activation	~	↑
LDL production	↑	↑
LDL oxidation	↓	↑
Ox-LDL/LOX-1 binding	↓	↑
Foam cell formation	↓	↑
Atypical angina	↑	↑
Responsiveness to antiplatelet drugs	↓	↓
Incidence of ABI <0.90	↑	↑
Ischemia area at cardiac stress imaging	↓	↑
Prevalence and severity of CAC	~	~
IMT	↓	↑

Legend: ↓, reduced; ↑, increased; ~, no differences; CAD, coronary artery disease; PAD, peripheral artery disease; AGEs, advanced glycation end-products; TLR, toll-like receptor; LDL low-density lipoproteins; Ox-LDL, oxidized low-density lipoprotein; LOX, lectin-like oxidized low-density lipoprotein receptor; ABI, ankle brachial index; CAC, coronary artery calcium; IMT, intima-media thickness.

## References

[1] European Heart Network (2017). European Cardiovascular Disease Statistics 2017.

[2] Maas A.H., Appelman Y.E. (2010). Gender differences in coronary heart disease. Neth. Heart J..

[3] Crilly M.A., Bundred P.E., Leckey L.C., Johnstone F.C. (2008). Gender bias in the clinical management of women with angina: Another look at the Yentl syndrome. J. Womens Health.

[4] Arora S., Stouffer G.A., Kucharska-Newton A., Vaduganathan M., Qamar A., Matsushita K., Kolte D., Reynolds H.R., Bangalore S., Rosamond W.D. (2018). Fifteen-Year Trends in Management and Outcomes of Non-ST-Segment-Elevation Myocardial Infarction Among Black and White Patients: The ARIC Community Surveillance Study, 2000–2014. J. Am. Heart Assoc..

[5] White U.A., Tchoukalova Y.D. (2014). Sex dimorphism and depot differences in adipose tissue function. Biochim. Biophys. Acta.

[6] Vijay V., Han T., Moland C.L., Kwekel J.C., Fuscoe J.C., Desai V.G. (2015). Sexual dimorphism in the expression of mitochondria-related genes in rat heart at different ages. PLoS ONE.

[7] International Diabetes Federation (2011). Diabetes Atlas.

[8] Masding M.G., Stears A.J., Burdge G.C., Wootton S.A., Sandeman D.D. (2003). Premenopausal advantages in postprandial lipid metabolism are lost in women with type 2 diabetes. Diabetes Care.

[9] Kannel W.B., Hjortland M., Castelli W.P. (1974). Role of diabetes in congestive heart failure: The Framingham study. Am. J. Cardiol..

[10] Ren J., Kelley R.O. (2009). Cardiac health in women with metabolic syndrome: Clinical aspects and pathophysiology. Obesity.

[11] De Rosa S., Chiefari E., Salerno N., Ventura V., D’Ascoli G.L., Arcidiacono B., Ambrosio G., Bilotta F.L., Torella D., Foti D. (2017). HMGA1 is a novel candidate gene for myocardial infarction susceptibility. Int. J. Cardiol..

[12] Barrett-Connor E.L., Cohn B.A., Wingard D.L., Edelstein S.L. (1991). Why is diabetes mellitus a stronger risk factor for fatal ischemic heart disease in women than in men? The Rancho Bernardo Study. JAMA.

[13] Barrett-Connor E., Bush T.L. (1991). Estrogen and coronary heart disease in women. JAMA.

[14] Manson J.E., Colditz G.A., Stampfer M.J., Willett W.C., Krolewski A.S., Rosner B., Arky R.A., Speizer F.E., Hennekens C.H. (1991). A prospective study of maturity-onset diabetes mellitus and risk of coronary heart disease and stroke in women. Arch. Intern. Med..

[15] Juutilainen A., Kortelainen S., Lehto S., Ronnemaa T., Pyorala K., Laakso M. (2004). Gender difference in the impact of type 2 diabetes on coronary heart disease risk. Diabetes Care.

[16] Huxley R., Barzi F., Woodward M. (2006). Excess risk of fatal coronary heart disease associated with diabetes in men and women: Meta-analysis of 37 prospective cohort studies. BMJ.

[17] Kiencke S., Handschin R., von Dahlen R., Muser J., Brunner-Larocca H.P., Schumann J., Felix B., Berneis K., Rickenbacher P. (2010). Pre-clinical diabetic cardiomyopathy: Prevalence, screening, and outcome. Eur. J. Heart Fail..

[18] Suys B.E., Katier N., Rooman R.P., Matthys D., Op De Beeck L., Du Caju M.V., De Wolf D. (2004). Female children and adolescents with type 1 diabetes have more pronounced early echocardiographic signs of diabetic cardiomyopathy. Diabetes Care.

[19] Huxley R.R., Peters S.A., Mishra G.D., Woodward M. (2015). Cardiovascular disease risk in type 1 diabetes—Authors’ reply. Lancet Diabetes Endocrinol..

[20] Huxley R.R., Peters S.A., Mishra G.D., Woodward M. (2015). Risk of all-cause mortality and vascular events in women versus men with type 1 diabetes: A systematic review and meta-analysis. Lancet Diabetes Endocrinol..

[21] Peters S., Smit E. (2014). Pan-HER inhibition in EGFR wild-type non-small-cell lung cancer. Lancet Oncol..

[22] Peters S.A., Huxley R.R., Woodward M. (2014). Diabetes as a risk factor for stroke in women compared with men: A systematic review and meta-analysis of 64 cohorts, including 775,385 individuals and 12,539 strokes. Lancet.

[23] Peters S.A., Huxley R.R., Sattar N., Woodward M. (2015). Sex Differences in the Excess Risk of Cardiovascular Diseases Associated with Type 2 Diabetes: Potential Explanations and Clinical Implications. Curr. Cardiovasc. Risk Rep..

[24] (2014). National Diabetes Audit–2012–2013 Report 1: Care Processes and Treatment Targets, Health and Social Care Information Centre 2014.

[25] Beckman J.A., Paneni F., Cosentino F., Creager M.A. (2013). Diabetes and vascular disease: Pathophysiology, clinical consequences, and medical therapy: Part II. Eur. Heart J..

[26] Evans R.W., Orchard T.J. (1994). Oxidized lipids in insulin-dependent diabetes mellitus: A sex-diabetes interaction?. Metab. Clin. Exp..

[27] Howard B.V., Cowan L.D., Go O., Welty T.K., Robbins D.C., Lee E.T. (1998). Adverse effects of diabetes on multiple cardiovascular disease risk factors in women. The Strong Heart Study. Diabetes Care.

[28] Mansfield M.W., Heywood D.M., Grant P.J. (1996). Sex differences in coagulation and fibrinolysis in white subjects with non-insulin-dependent diabetes mellitus. Arterioscler. Thromb. Vasc. Biol..

[29] Ossei-Gerning N., Wilson I.J., Grant P.J. (1998). Sex differences in coagulation and fibrinolysis in subjects with coronary artery disease. Thromb. Haemost..

[30] Steinberg H.O., Paradisi G., Cronin J., Crowde K., Hempfling A., Hook G., Baron A.D. (2000). Type II diabetes abrogates sex differences in endothelial function in premenopausal women. Circulation.

[31] Wannamethee S.G., Papacosta O., Lawlor D.A., Whincup P.H., Lowe G.D., Ebrahim S., Sattar N. (2012). Do women exhibit greater differences in established and novel risk factors between diabetes and non-diabetes than men? The British Regional Heart Study and British Women’s Heart Health Study. Diabetologia.

[32] Garaulet M., Perex-Llamas F., Fuente T., Zamora S., Tebar F.J. (2000). Anthropometric, computed tomography and fat cell data in an obese population: Relationship with insulin, leptin, tumor necrosis factor-alpha, sex hormone-binding globulin and sex hormones. Eur. J. Endocrinol..

[33] Donahue R.P., Rejman K., Rafalson L.B., Dmochowski J., Stranges S., Trevisan M. (2007). Sex differences in endothelial function markers before conversion to pre-diabetes: Does the clock start ticking earlier among women? The Western New York Study. Diabetes Care.

[34] Haffner S.M., Miettinen H., Stern M.P. (1997). Relatively more atherogenic coronary heart disease risk factors in prediabetic women than in prediabetic men. Diabetologia.

[35] Bugger H., Abel E.D. (2009). Rodent models of diabetic cardiomyopathy. Dis. Models Mech..

[36] Bhatia A., Sekhon H.K., Kaur G. (2014). Sex hormones and immune dimorphism. Sci. World J..

[37] Balistreri C.R., Accardi G., Candore G. (2013). Probiotics and Prebiotics: Health Promotion by Immune Modulation in the Elderly.

[38] Kander M.C., Cui Y., Liu Z. (2017). Gender difference in oxidative stress: A new look at the mechanisms for cardiovascular diseases. J. Cell. Mol. Med..

[39] Garaulet M., Perez-Llamas F., Baraza J.C., Garcia-Prieto M.D., Fardy P.S., Tebar F.J., Zamora S. (2002). Body fat distribution in pre-and post-menopausal women: Metabolic and anthropometric variables. J. Nutr. Health Aging.

[40] Lima R., Wofford M., Reckelhoff J.F. (2012). Hypertension in postmenopausal women. Curr. Hypertens. Rep..

[41] Link J.C., Chen X., Arnold A.P., Reue K. (2013). Metabolic impact of sex chromosomes. Adipocyte.

[42] Link J.C., Reue K. (2017). Genetic Basis for Sex Differences in Obesity and Lipid Metabolism. Annu. Rev. Nutr..

[43] Zore T., Palafox M., Reue K. (2018). Sex differences in obesity, lipid metabolism, and inflammation-A role for the sex chromosomes?. Mol. Metab..

[44] Turnbaugh P.J., Backhed F., Fulton L., Gordon J.I. (2008). Diet-induced obesity is linked to marked but reversible alterations in the mouse distal gut microbiome. Cell Host Microbe.

[45] Dehghan P., Gargari B.P., Jafar-Abadi M.A., Aliasgharzadeh A. (2014). Inulin controls inflammation and metabolic endotoxemia in women with type 2 diabetes mellitus: A randomized-controlled clinical trial. Int. J. Food Sci. Nutr..

[46] Sinha T., Vich Vila A., Garmaeva S., Jankipersadsing S.A., Imhann F., Collij V., Bonder M.J., Jiang X., Gurry T., Alm E.J. (2018). Analysis of 1135 gut metagenomes identifies sex-specific resistome profiles. Gut Microbes.

[47] Vemuri R., Sylvia K.E., Klein S.L., Forster S.C., Plebanski M., Eri R., Flanagan K.L. (2018). The microgenderome revealed: Sex differences in bidirectional interactions between the microbiota, hormones, immunity and disease susceptibility. Semin. Immunopathol..

[48] Aravindhan V., Madhumitha H. (2016). Metainflammation in Diabetic Coronary Artery Disease: Emerging Role of Innate and Adaptive Immune Responses. J. Diabetes Res..

[49] Wang M., Monticone R.E., Lakatta E.G. (2014). Proinflammation of aging central arteries: A mini-review. Gerontology.

[50] Lontchi-Yimagou E., Sobngwi E., Matsha T.E., Kengne A.P. (2013). Diabetes mellitus and inflammation. Curr. Diabetes Rep..

[51] Hotamisligil G.S. (2017). Inflammation, metaflammation and immunometabolic disorders. Nature.

[52] Kim S.H., Lee J.W., Im J.A., Hwang H.J. (2011). Monocyte chemoattractant protein-1 is related to metabolic syndrome and homocysteine in subjects without clinically significant atherosclerotic cardiovascular disease. Scand. J. Clin. Lab. Investig..

[53] Jia S.J., Niu P.P., Cong J.Z., Zhang B.K., Zhao M. (2014). TLR4 signaling: A potential therapeutic target in ischemic coronary artery disease. Int. Immunopharmacol..

[54] Balistreri C.R., Ruvolo G., Lio D., Madonna R. (2017). Toll-like receptor-4 signaling pathway in aorta aging and diseases: “its double nature”. J. Mol. Cell. Cardiol..

[55] Yamagishi S., Fukami K., Matsui T. (2015). Crosstalk between advanced glycation end products (AGEs)-receptor RAGE axis and dipeptidyl peptidase-4-incretin system in diabetic vascular complications. Cardiovasc. Diabetol..

[56] Madonna R., Balistreri C.R., Geng Y.J., De Caterina R. (2017). Diabetic microangiopathy: Pathogenetic insights and novel therapeutic approaches. Vasc. Pharmacol..

[57] Velloso L.A., Folli F., Saad M.J. (2015). TLR4 at the Crossroads of Nutrients, Gut Microbiota, and Metabolic Inflammation. Endocr. Rev..

[58] Orsatti C.L., Petri Nahas E.A., Nahas-Neto J., Orsatti F.L., Giorgi V.I., Witkin S.S. (2014). Evaluation of Toll-Like receptor 2 and 4 RNA expression and the cytokine profile in postmenopausal women with metabolic syndrome. PLoS ONE.

[59] Balistreri C.R., Bonfigli A.R., Boemi M., Olivieri F., Ceriello A., Genovese S., Franceschi C., Spazzafumo L., Fabietti P., Candore G. (2014). Evidences of +896 A/G TLR4 polymorphism as an indicative of prevalence of complications in T2DM patients. Med. Inflamm..

[60] Matthan N.R., Jalbert S.M., Barrett P.H., Dolnikowski G.G., Schaefer E.J., Lichtenstein A.H. (2008). Gender-specific differences in the kinetics of nonfasting TRL, IDL, and LDL apolipoprotein B-100 in men and premenopausal women. Arterioscler. Thromb. Vasc. Biol..

[61] Magkos F., Patterson B.W., Mohammed B.S., Klein S., Mittendorfer B. (2007). Women produce fewer but triglyceride-richer very low-density lipoproteins than men. J. Clin. Endocrinol. Metab..

[62] Hewitt K.N., Boon W.C., Murata Y., Jones M.E., Simpson E.R. (2003). The aromatase knockout mouse presents with a sexually dimorphic disruption to cholesterol homeostasis. Endocrinology.

[63] Sakurai K., Sawamura T. (2003). Stress and vascular responses: Endothelial dysfunction via lectin-like oxidized low-density lipoprotein receptor-1: Close relationships with oxidative stress. J. Pharmacol. Sci..

[64] Mehta J.L., Chen J., Hermonat P.L., Romeo F., Novelli G. (2006). Lectin-like, oxidized low-density lipoprotein receptor-1 (LOX-1): A critical player in the development of atherosclerosis and related disorders. Cardiovasc. Res..

[65] Brownlee M. (2001). Biochemistry and molecular cell biology of diabetic complications. Nature.

[66] Kork F., Jankowski V., Just A.R., Pfeilschifter J., Tepel M., Zidek W., Jankowski J. (2014). Oxidized low-density lipoprotein in postmenopausal women. J. Hypertens..

[67] Brinkley T.E., Kume N., Mitsuoka H., Phares D.A., Hagberg J.M. (2008). Elevated soluble lectin-like oxidized LDL receptor-1 (sLOX-1) levels in obese postmenopausal women. Obesity.

[68] Bukowska A., Spiller L., Wolke C., Lendeckel U., Weinert S., Hoffmann J., Bornfleth P., Kutschka I., Gardemann A., Isermann B. (2017). Protective regulation of the ACE2/ACE gene expression by estrogen in human atrial tissue from elderly men. Exp. Biol. Med..

[69] Kume N., Murase T., Moriwaki H., Aoyama T., Sawamura T., Masaki T., Kita T. (1998). Inducible expression of lectin-like oxidized LDL receptor-1 in vascular endothelial cells. Circ. Res..

[70] Chen M., Nagase M., Fujita T., Narumiya S., Masaki T., Sawamura T. (2001). Diabetes enhances lectin-like oxidized LDL receptor-1 (LOX-1) expression in the vascular endothelium: Possible role of LOX-1 ligand and AGE. Biochem. Biophys. Res. Commun..

[71] Garrido-Sanchez L., Cardona F., Garcia-Fuentes E., Rojo-Martinez G., Gomez-Zumaquero J.M., Picon M.J., Soriguer F., Tinahones F.J. (2008). Anti-oxidized low-density lipoprotein antibody levels are associated with the development of type 2 diabetes mellitus. Eur. J. Clin. Investig..

[72] Cominacini L., Rigoni A., Pasini A.F., Garbin U., Davoli A., Campagnola M., Pastorino A.M., Lo Cascio V., Sawamura T. (2001). The binding of oxidized low density lipoprotein (ox-LDL) to ox-LDL receptor-1 reduces the intracellular concentration of nitric oxide in endothelial cells through an increased production of superoxide. J. Biol. Chem..

[73] Bertoluci M.C., Ce G.V., da Silva A.M., Wainstein M.V., Boff W., Punales M. (2015). Endothelial dysfunction as a predictor of cardiovascular disease in type 1 diabetes. World J. Diabetes.

[74] Meischke H., Larsen M.P., Eisenberg M.S. (1998). Gender differences in reported symptoms for acute myocardial infarction: Impact on prehospital delay time interval. Am. J. Emerg. Med..

[75] Khan N.A., Daskalopoulou S.S., Karp I., Eisenberg M.J., Pelletier R., Tsadok M.A., Dasgupta K., Norris C.M., Pilote L., Genesis Praxy Team (2013). Sex differences in acute coronary syndrome symptom presentation in young patients. JAMA Intern. Med..

[76] Shah A.S., Griffiths M., Lee K.K., McAllister D.A., Hunter A.L., Ferry A.V., Cruikshank A., Reid A., Stoddart M., Strachan F. (2015). High sensitivity cardiac troponin and the under-diagnosis of myocardial infarction in women: Prospective cohort study. BMJ.

[77] Levin R.I. (2005). The puzzle of aspirin and sex. N. Engl. J. Med..

[78] Kuller L., Borhani N., Furberg C., Gardin J., Manolio T., O’Leary D., Psaty B., Robbins J. (1994). Prevalence of subclinical atherosclerosis and cardiovascular disease and association with risk factors in the Cardiovascular Health Study. Am. J. Epidemiol..

[79] Karam N., Marijon E., Bougouin W., Spaulding C., Jouven X. (2016). [Sudden cardiac death: Are women different?]. Annales de Cardiologie et D’angeiologie..

[80] Berthome P., Tixier R., Briand J., Geoffroy O., Babuty D., Mansourati J., Jesel L., Dupuis J.M., Bru P., Kyndt F. (2018). Clinical presentation and follow-up of women affected by Brugada syndrome. Heart Rhythm.

[81] Catalan M., Herreras Z., Pinyol M., Sala-Vila A., Amor A.J., de Groot E., Gilabert R., Ros E., Ortega E. (2015). Prevalence by sex of preclinical carotid atherosclerosis in newly diagnosed type 2 diabetes. Nutrition, metabolism, and cardiovascular diseases. NMCD.

[82] Reynolds H.R., Hausvater A., Carney K. (2018). Test Selection for Women with Suspected Stable Ischemic Heart Disease. J. Womens Health.

[83] Pathak L.A., Shirodkar S., Ruparelia R., Rajebahadur J. (2017). Coronary artery disease in women. Indian Heart J..

[84] Irace C., De Rosa S., Tripolino C., Ambrosio G., Covello C., Abramo E., Carallo C., Mongiardo A., Spaccarotella C., Torella D. (2018). Delayed flow-mediated vasodilation and critical coronary stenosis. J. Investig. Med..

[85] Aboyans V., Criqui M.H., McClelland R.L., Allison M.A., McDermott M.M., Goff D.C., Manolio T.A. (2007). Intrinsic contribution of gender and ethnicity to normal ankle-brachial index values: The Multi-Ethnic Study of Atherosclerosis (MESA). J. Vasc. Surg..

[86] Igarashi Y., Chikamori T., Hida S., Tanaka H., Shiba C., Usui Y., Hatano T., Yamashina A. (2011). Importance of the ankle-brachial pressure index in the diagnosis of coronary artery disease in women with diabetes without anginal pain. Circ. J..

[87] Raggi P., Gongora M.C., Gopal A., Callister T.Q., Budoff M., Shaw L.J. (2008). Coronary artery calcium to predict all-cause mortality in elderly men and women. J. Am. Coll. Cardiol..

[88] Wackers F.J., Young L.H., Inzucchi S.E., Chyun D.A., Davey J.A., Barrett E.J., Taillefer R., Wittlin S.D., Heller G.V., Filipchuk N. (2004). Detection of silent myocardial ischemia in asymptomatic diabetic subjects: The DIAD study. Diabetes Care.

[89] Berman D.S., Kang X., Hayes S.W., Friedman J.D., Cohen I., Abidov A., Shaw L.J., Amanullah A.M., Germano G., Hachamovitch R. (2003). Adenosine myocardial perfusion single-photon emission computed tomography in women compared with men. Impact of diabetes mellitus on incremental prognostic value and effect on patient management. J. Am. Coll. Cardiol..

[90] Mielke C.H., Shields J.P., Broemeling L.D. (2001). Coronary artery calcium, coronary artery disease, and diabetes. Diabetes Res. Clin. Pract..

[91] Schindler T.H., Zhang X.L., Vincenti G., Mhiri L., Lerch R., Schelbert H.R. (2007). Role of PET in the evaluation and understanding of coronary physiology. J. Nucl. Cardiol..

[92] Camici P.G., Crea F. (2007). Coronary microvascular dysfunction. N. Engl. J. Med..

[93] Bengel F.M., Higuchi T., Javadi M.S., Lautamaki R. (2009). Cardiac positron emission tomography. J. Am. Coll. Cardiol..

[94] Camici P.G., Rimoldi O.E. (2009). The clinical value of myocardial blood flow measurement. J. Nucl. Med..

[95] Ganz P., Vita J.A. (2003). Testing endothelial vasomotor function: Nitric oxide, a multipotent molecule. Circulation.

[96] Naya M., Tsukamoto T., Morita K., Katoh C., Furumoto T., Fujii S., Tamaki N., Tsutsui H. (2007). Olmesartan, but not amlodipine, improves endothelium-dependent coronary dilation in hypertensive patients. J. Am. Coll. Cardiol..

[97] Sato H., Fujimoto S., Kogure Y., Daida H. (2018). Feasibility of Macrophage Plaque Imaging Using Novel Ultrasmall Superparamagnetic Iron Oxide in Dual Energy CT. Eur. J. Radiol. Open.

[98] Sampson U.K., Dorbala S., Limaye A., Kwong R., Di Carli M.F. (2007). Diagnostic accuracy of rubidium-82 myocardial perfusion imaging with hybrid positron emission tomography/computed tomography in the detection of coronary artery disease. J. Am. Coll. Cardiol..

[99] Bateman T.M., Heller G.V., McGhie A.I., Friedman J.D., Case J.A., Bryngelson J.R., Hertenstein G.K., Moutray K.L., Reid K., Cullom S.J. (2006). Diagnostic accuracy of rest/stress ECG-gated Rb-82 myocardial perfusion PET: Comparison with ECG-gated Tc-99m sestamibi SPECT. J. Nucl. Cardiol..

[100] Marchesseau S., Seneviratna A., Sjoholm A.T., Qin D.L., Ho J.X.M., Hausenloy D.J., Townsend D.W., Richards A.M., Totman J.J., Chan M.Y.Y. (2018). Hybrid PET/CT and PET/MRI imaging of vulnerable coronary plaque and myocardial scar tissue in acute myocardial infarction. J. Nucl. Cardiol..

[101] Senders M.L., Hernot S., Carlucci G., van de Voort J.C., Fay F., Calcagno C., Tang J., Alaarg A., Zhao Y., Ishino S. (2018). Nanobody-Facilitated Multiparametric PET/MRI Phenotyping of Atherosclerosis. JACC Cardiovasc. Imaging.

[102] Basso C., Perazzolo Marra M., Rizzo S., De Lazzari M., Giorgi B., Cipriani A., Frigo A.C., Rigato I., Migliore F., Pilichou K. (2015). Arrhythmic Mitral Valve Prolapse and Sudden Cardiac Death. Circulation.

[103] Larsen J.R., Brekke M., Bergengen L., Sandvik L., Arnesen H., Hanssen K.F., Dahl-Jorgensen K. (2005). Mean HbA1c over 18 years predicts carotid intima media thickness in women with type 1 diabetes. Diabetologia.

[104] Pithova P., Stechova K., Pitha J., Lanska V., Kvapil M. (2016). Determinants of preclinical atherosclerosis are different in type 1 and type 2 diabetic women. Physiol. Res..

[105] Woodard G.A., Brooks M.M., Barinas-Mitchell E., Mackey R.H., Matthews K.A., Sutton-Tyrrell K. (2011). Lipids, menopause, and early atherosclerosis in Study of Women’s Health Across the Nation Heart women. Menopause.

[106] Polotsky H.N., Polotsky A.J. (2010). Metabolic implications of menopause. Semin. Reprod. Med..

[107] Sanchez-Inigo L., Navarro-Gonzalez D., Fernandez-Montero A., Pastrana-Delgado J., Martinez J.A. (2016). The TyG index may predict the development of cardiovascular events. Eur. J. Clin. Investig..

[108] Lambrinoudaki I., Kazani M.V., Armeni E., Georgiopoulos G., Tampakis K., Rizos D., Augoulea A., Kaparos G., Alexandrou A., Stamatelopoulos K. (2018). The TyG Index as a Marker of Subclinical Atherosclerosis and Arterial Stiffness in Lean and Overweight Postmenopausal Women. Heart Lung Circ..

[109] De Rosa S., Arcidiacono B., Chiefari E., Brunetti A., Indolfi C., Foti D.P. (2018). Type 2 Diabetes Mellitus and Cardiovascular Disease: Genetic and Epigenetic Links. Front. Endocrinol..

[110] Jones D.A., Prior S.L., Tang T.S., Bain S.C., Hurel S.J., Humphries S.E., Stephens J.W. (2010). Association between the rs4880 superoxide dismutase 2 (C>T) gene variant and coronary heart disease in diabetes mellitus. Diabetes Res. Clin. Pract..

[111] Vendrell J., Fernandez-Real J.M., Gutierrez C., Zamora A., Simon I., Bardaji A., Ricart W., Richart C. (2003). A polymorphism in the promoter of the tumor necrosis factor-alpha gene (-308) is associated with coronary heart disease in type 2 diabetic patients. Atherosclerosis.

[112] Chan K.H., Huang Y.T., Meng Q., Wu C., Reiner A., Sobel E.M., Tinker L., Lusis A.J., Yang X., Liu S. (2014). Shared molecular pathways and gene networks for cardiovascular disease and type 2 diabetes mellitus in women across diverse ethnicities. Circulation. Cardiovasc. Genet..

